# Selectively Patterning Polymer Opal Films via Microimprint Lithography

**DOI:** 10.1002/adom.201400327

**Published:** 2014-09-01

**Authors:** Tao Ding, Qibin Zhao, Stoyan K Smoukov, Jeremy J Baumberg

**Affiliations:** Nanophotonic Centre Cavendish Laboratory University of CambridgeCB3 0HE, UK E-mail: jjb12@cam.ac.uk; Department of Materials Science and Metallurgy27 Charles Babbage RoadUniversity of CambridgeCB3 0FS, UK

**Keywords:** shear flow, core–shell particles, photonic crystals, structural color, polymer opal films, lithography

## Abstract

Large-scale structural color flexible coatings have been hard to create, and patterning color on them is key to many applications, including large-area strain sensors, wall-size displays, security devices, and smart fabrics. To achieve controlled tuning, a micro-imprinting technique is applied here to pattern both the surface morphology and the structural color of the polymer opal films (POFs). These POFs are made of 3D ordered arrays of hard spherical particles embedded inside soft shells. The soft outer shells cause the POFs to deform upon imprinting with a pre-patterned stamp, driving a flow of the soft polymer and a rearrangement of the hard spheres within the films. As a result, a patterned surface morphology is generated within the POFs and the structural colors are selectively modified within different regions. These changes are dependent on the pressure, temperature, and duration of imprinting, as well as the feature sizes in the stamps. Moreover, the pattern geometry and structural colors can then be further tuned by stretching. Micropattern color generation upon imprinting depends on control of colloidal transport in a polymer matrix under shear flow and brings many potential properties including stretchability and tunability, as well as being of fundamental interest.

## 1. Introduction

Photonic crystals are periodic structures on the scale of the optical wavelength, which control the flow of light, and can produce strong optical effects based on their nanostructure. Patterning of photonic crystals has been an intriguing and timely topic among material scientists seeking structural color applications, with several rigorous studies over the last decade.[1–20] By controlling the placement of photonic crystal patterns, intricate photonic circuits and devices can be developed for potential optical computation and communication systems.[2,3] Beyond this, photonic crystal patterns can be used as active sensors and image displays,[4–8] which can meet the strong demand for smart functional materials.

Although many different approaches have been developed for the patterning of photonic crystals, it remains a great challenge to cost-effectively achieve large-scale photonic patterns which can be actively tuned. Traditional ways of patterning photonic crystals involve lithography,[9] self-assembly[10] or template directed approaches.[11–20] However, most of these methods are either complicated and time-consuming, or the patterns are too simple with no tunability. While inkjet printing provides a promising approach towards sequentially written colloidal photonic crystal patterns, the droplets must be well separated, the surface hydrophicity needs to be highly controlled to ensure crystallization, and they form highly convex domes which are written only one at a time.[21–23] Recent efforts have also been devoted to generating complex and tunable colloidal photonic crystal patterns controlled by external stimuli such as electric and magnetic fields or chemicals.[4–8]

In this paper, we report mechanically tunable patterns generated via a microimprinting technique. Micro/nanoimprinting is renowned for its wafer-scale patterning with good reproducibility and fidelity.[24] However, this high utility patterning technique has been hard to apply to pattern 3D photo­nic crystals. The main challenges are the high melting temperature required for imprinting (which always destroys the ordered photonic nanostructures), and that photonic crystals made of inorganics are too rigid to be imprinted by any stamps. Recently we have developed polymer opals films (POFs) made of poly(styrene-methyl acrylate-ethyl acrylate) (PS@PMMA@PEA) colloidal arrays.[25–27] The cross-linked PS cores (with higher *T*_g_) are rigid and covalently bonded to outer soft PEA (low *T*_g_) shells. Therefore at appropriate temperatures the PEA shells melt into a ‘solvent’ matrix and drive the flow of PS spheres under directional shearing forces which generate ordered arrays of opal-like nano-architecture possessing iridescent structural colors. Such soft and rubbery POFs provided a perfect system for microimprint patterning. The imprint lithography not only generates different structural color patterns within the POFs but also induces localized shear ordering of microdomains perpendicular to the film. The mechanism is thus very different from our previous reports of horizontal shearing to obtain mass-scale ordering, and should guide refinements of modeling such viscous colloidal shear flows.

**Scheme 1:**
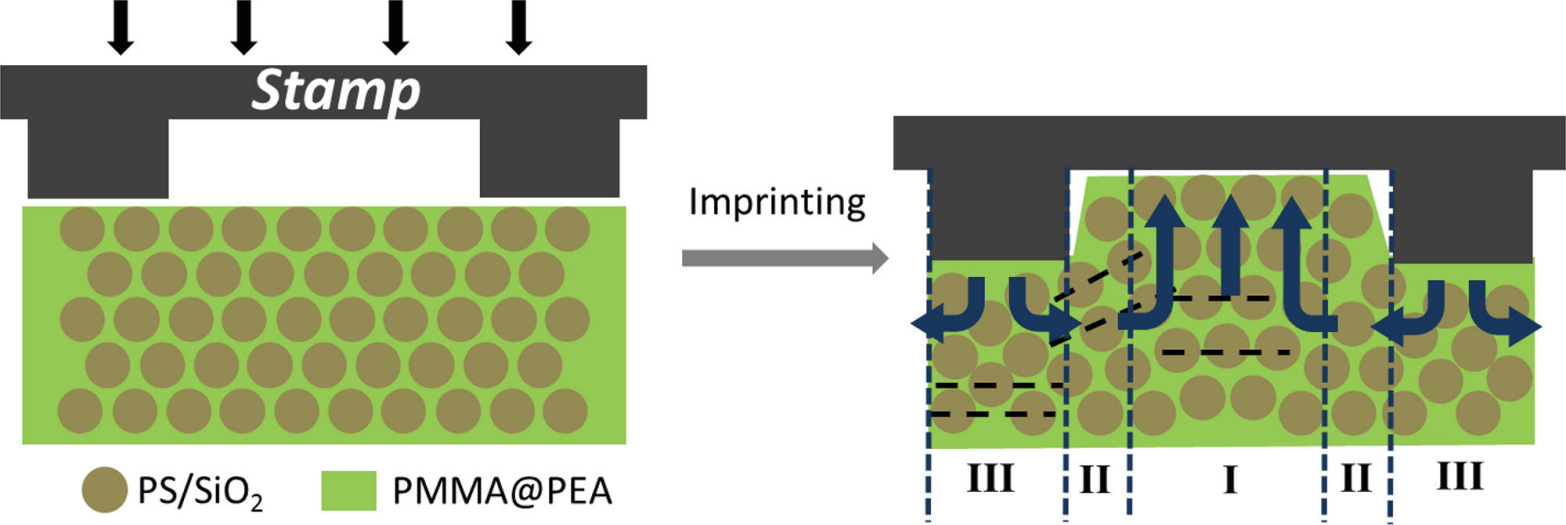
Mechanism of pattern formation by microimprinting in POFs. Region I is the extruded region, region II is the transition region, and region III is the imprinted region.

We start from the fabrication of POFs of uniform color as previously reported, using a well-developed shear-ordering process.[27] Upon micro-imprinting these solid flexible films, the PEA matrix containing the hard spherical particles deforms into negative patterns of the stamp resulting in a rearrangement of the spheres as shown in **Scheme** **1**. The intrusion of the hard posts squeezes the POF laterally into the void between neighboring posts of the stamp. Such lateral expulsion leads to expanded sphere separations in region I. Simultaneously, the compression directly under the stamp mesa decreases the vertical distance between neighboring sphere layers in region III. In between regions I and III is a transition region II, where most of the shearing forces in the vertical direction are concentrated, which results in a local distortion of the sphere lattice. Although shearing in this fashion is detrimental to an ordered lattice, it can actually align randomly arranged spherical particles into ordered arrays as we show below. Because of the different geometry of the colloidal lattice within these three regions, the uniform structural color of the original POF splits into patterns of three different colors with different intensity. Since the color patterns are dictated by the flow of the PEA matrix then different separations of the posts, pressures or temperatures result in different color patterns. The rubbery properties of the POFs allow these patterns of structural colors and their geometry to be fine-tuned via mechanical stretching. Because of the scalability and tunability of such POF patterns, they promise applications in active sensing, low-power large-area image displays, and anti-fraud and anti-counterfeit materials.

## 2. Results and Discussion

Heterogenous nanoparticles made of hard cores and soft shells are critical for the shearing-induced self-assembly of the POFs.[25] The soft PEA layer with low *T*_g_ can melt and flow to form an elastic matrix upon extrusion and shearing. The hard cores, which are here made of either heavily cross-linked PS or silica, are bonded to the matrix PEA and can align and arrange themselves into ordered arrays upon repeated directional shearing.[27] Such soft and elastic POFs are robust enough for active tuning through stretching, squeezing, compression and bending.[28] Combining opals with imprinting techniques, we develop potential for sophisticated functional patterning of such POFs.

### 2.1. Microimprinting of Polymer Opal Films

**Figure 1:**
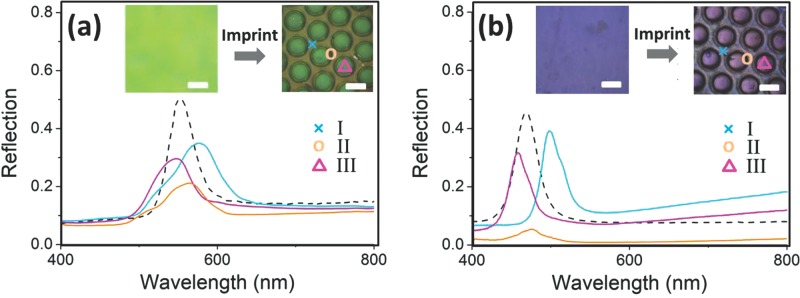
Reflection spectra of POFs before (dashed) and after (solid) the imprinting process. Line colors show marked regions of inset optical images. Scale bars in insets are 100 μm. (a) POF made of PS@PMMA@PEA, (b) POF made of SiO_2_@PMMA@PEA.

The stamps used for imprinting are made by replicating a PDMS mold with epoxy resin. The initial stamp used comprises of a hexagonal array of 30 μm high posts with diameter 100 μm and intervening gap of 50 μm (see SI Figure S1). The standard imprinting process used 30 bar pressure at 110 °C for 150 s, controlled by an Obducat Nanoimprinter. We patterned 100 μm thick POFs built of PS@PMMA@PEA (diameter 230 nm) and SiO_2_@PMMA@PEA (diameter 175 nm), with initial appearance and reflection spectra shown in **Figure** **1**. The PS@PMMA@PEA POFs initially have a bright green structural color with an intense reflection peak at 552 nm (Figure 1a dashed line and inset). After imprinting, distinctive color patterns are generated (Figure 1a inset). Three typical regions in this pattern are seen, corresponding to the squeezed, sheared, and compressed, regions (I-III), and marked with blue cross (×), orange circle (○), and purple triangle (▵) respectively in the image. Reflection spectra recorded confocally on these three regions (Figure 1a, solid lines of corresponding colors) capture these deformations. The resonant wavelengths of the compressed and squeezed regions are blue-shifted to 546 nm and red-shifted to 575 nm respectively, compared with the original peak at 552 nm. This is mainly because of a decrease of lattice spacing on compression and an increase of lattice spacing on sideways extrusion. The peak reflectivities drop to ∼30%, which may arise because of non-homogeneous flows or the introduction of defects during squeezing and compression. For the sheared region II, squeezing might be expected to expand the lattice spacing vertically, but although the reflection peak red-shifts slightly to 560 nm, the shearing forces instead introduce distortions and defects in this region, resulting in a further decrease of resonant reflection intensity to 20%.

Similar patterning behavior is found in the SiO_2_@PMMA@PEA system (Figure 1b). The imprinting process changes the original blue color (469 nm) into purple and cyan, located at 458 and 498 nm for regions III and I respectively, with only a small dimming of the reflection peaks. Again in the shear-flow region II, the reflection peak red-shifts slightly to 476 nm but the intensity drops dramatically. These phenomena are consistent with our proposed color patterning mechanism in Scheme 1.

**Figure 2:**
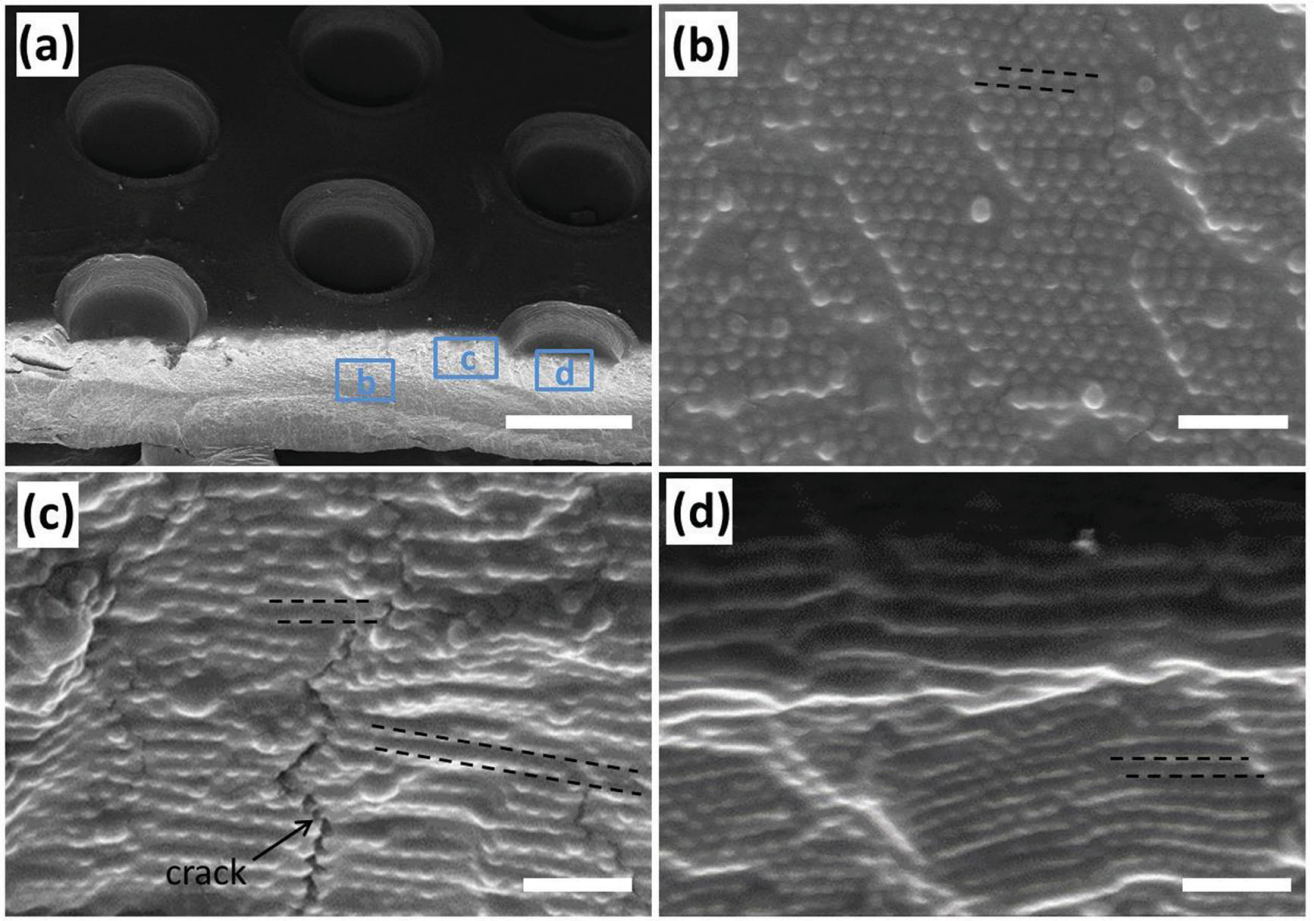
SEM images of the micropatterned POFs. (a) Angled view of patterned POFs made of SiO_2_@PMMA@PEA, scale bar is 100 μm. (b-c) Magnified views of framed regions in (a): Cross-section of (b) squeezed (I), (c) sheared (II), and (d) imprinted (III) regions. All scale bars are 1 μm.

To confirm this, more detailed inspection of the nanostructures was carried out using SEM (**Figure** **2**). For straightforward imaging of the samples, we preferred the SiO_2_@PMMA@PEA system because of its better contrast between SiO_2_ and polymer matrix. Tilted micropattern views of the hexagonal arrays of circular holes (Figure 2a) show that the imprinted holes are not fully embossed through the film by the posts, as expected from the imprint post height. The hard silica spheres in the composite are incompressible and hinder the free flow of PEA matrix underneath the stamp. Therefore the profile of the surface patterns is mainly due to compression of the imprinted regions and expansion of the neighboring regions. Magnified images of the squeezed (I), sheared (II) and compressed (III) regions (Figures 2b-d) reveal the ordered layers of SiO_2_ spheres as well as defects, which can be compared with the cross-section of the POF before imprinting (Figure S2). Initially the lattice plane spacing along the film normal is 128 nm, which increases after imprinting to 165 nm in region b (I) but decreases to 118 nm in region d (III) due to compression. The sheared region c (II) also showed an increase of lattice distance to 150 nm and the shear forces lead to distortion and cracks in the lattice as indicated in Figure 2c (although not tilting), which is the main reason for the drop of the reflection intensity.

### 2.2. Shear-induced Local Ordering by Microimprinting

**Figure 3:**
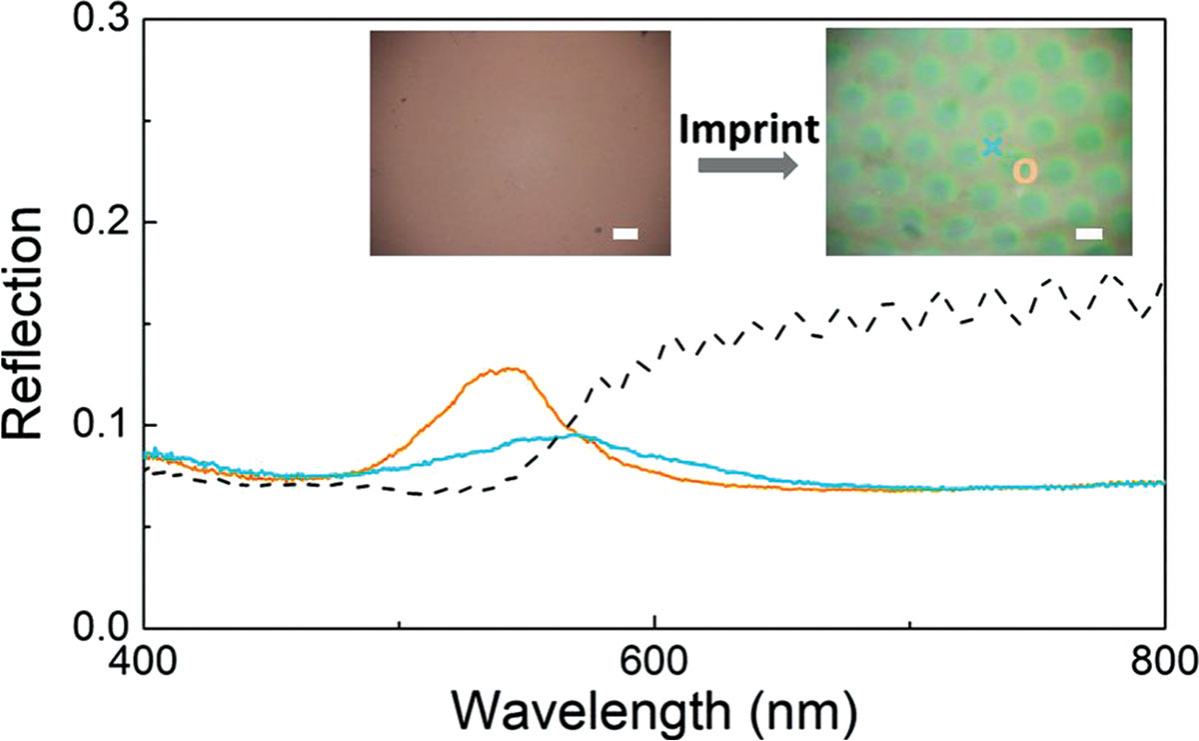
Reflection spectra of unordered (dashed) and imprinted POFs (sheared regions: orange solid line; squeezed regions: blue solid line). Insets show optical images of patterns (spectral measurements at points marked). Scale bar 100 μm.

So far, the shearing forces have reduced the order of the films rather than enhancing it. This is mainly because the shearing force imposed by imprinting is perpendicular to the in-plane shear originally used to order the POFs. Therefore it misaligns the lattice and introduces disorder. However if the core-shell nanoparticles are not pre-ordered initially, imprinting can actually result in the introduction of order along the shearing zones as shown in **Figure** **3**. Before imprinting, the POFs show poor structural color with almost no reflection peak (showing only scattering at short wavelengths). After imprinting, green colors are generated at the sheared regions with reflection peaks at 541 nm and a peak height of 7%, compared to 2% from the weakly sheared regions. The detailed SEM images in SI Figure S3 show that the locally sheared region induces partial ordering (Figure S3c) compared with the area before imprinting (Figure S3a). For the weakly sheared regions (Figure S3d), the ordering is significantly less effective with only a 2% reflection peak. Although this reflection peak is not yet comparable with POFs made by repeated horizontal shearing (up to *R* = 40% from 20 passes) shown in Figure 1, it provides a promising alternative and local way to produce small-scale ordering of colloidal assemblies which generates photonic crystal patterns. However in the rest of this paper we examine in more detail the mechanism of single-iteration imprints on initially ordered opal films to better understand and control the process for enhanced ordering and hence local structural color.

### 2.3. Tunable Structural Color Patterns by Control of Microimprinting Conditions

**Figure 4:**
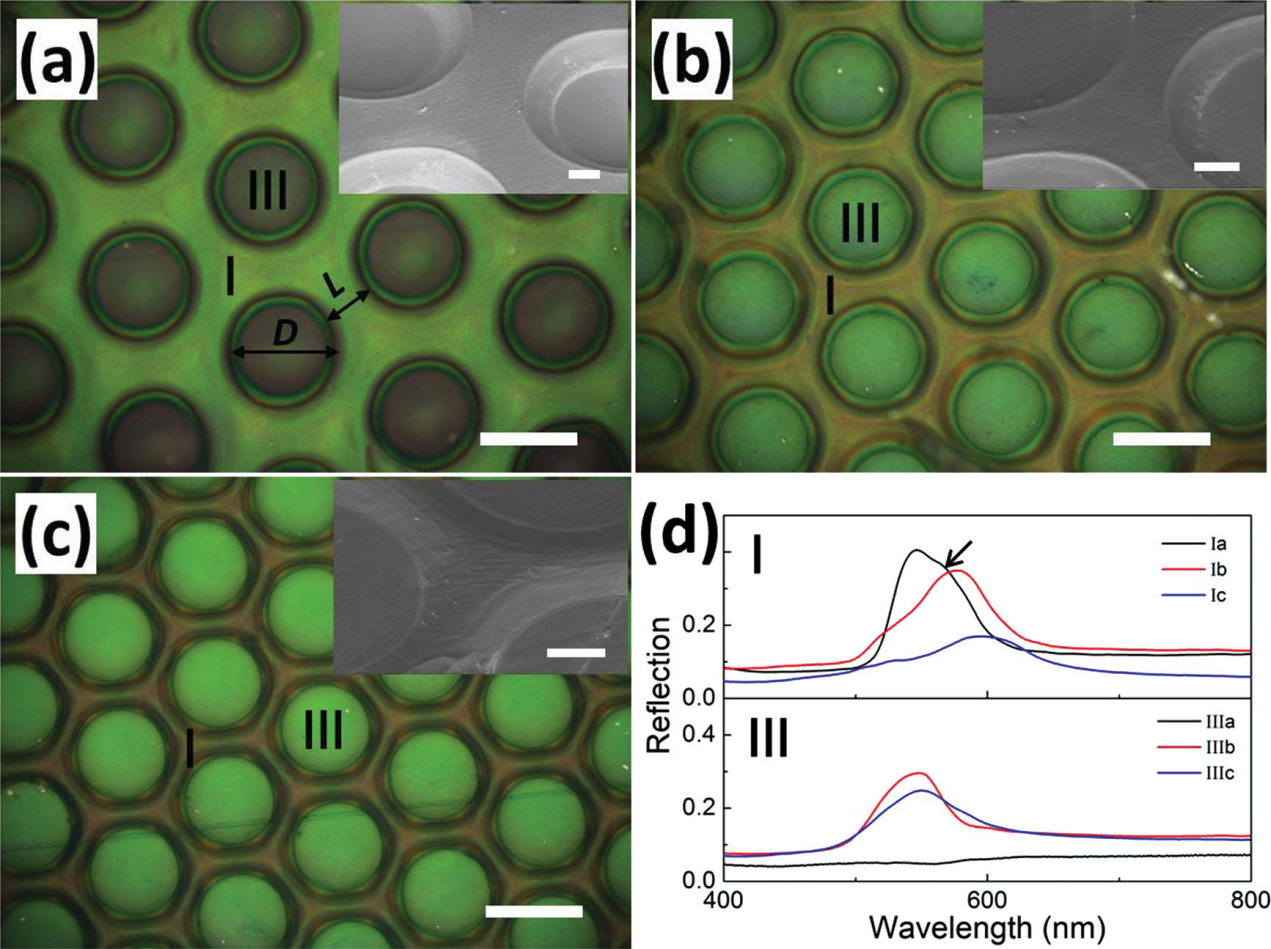
(a–c) Optical images of imprinted micropatterns on POFs with stamps of different post separation, *L* = 80, 50, 30 μm. Scale bars are 100 μm and 20 μm in insets. (d) Reflection spectra of patterns in (a–c) within regions I and III. Arrow indicates shoulder peak.

The changes in the structural colors observed are mainly determined by the flow properties of the polymer constituents. The imprinting depth *h* can be quantitatively estimated through[29]

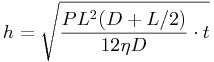
(1)

where *P* is the imprinting pressure, *D* is the diameter of the post, *L* is the gap width between the posts, *η* is the viscosity of the polymer matrix, and *t* is the imprinting time. This predicts that the more the polymer matrix flows, the deeper will be imprinted by the stamp. Therefore we tune the colors of the patterns produced through shear by adjusting the spacing (*L*) between the posts. Stamps with post separations of *L* = 80, 50, and 30 μm are used with the standard imprinting process on POFs made of PS@PMMA@PEA (**Figure** **4**). As predicted by Equation (1), smaller post separations allow shallower intrusion into the POFs by the post (giving holes of 16, 11 and 8 μm measured from the inset SEM images of Figures 4a–c respectively). The pressure imposed by the posts drives the lateral flow of PEA together with the PS cores to which it is covalently linked, resulting in the red-shift of color in the neighboring regions into which they are extruded. For the directly imprinted regions (III), different post separations produce different imprinting depth with the greater compression at large *L* increasing the flow from under each post and which, beyond a critical value, completely destroys the original order (Figures 4a,d III and SI-Figure S4). On the other hand, the extruded regions show much dimmer color when the separations are small (Figures 4c, d I), and the non-uniformities introduced are evident in the splitting of the reflectivity into two peaks, one of which red-shifts and other blue-shifts as *L* decreases (Figure 4d I). The induced red-shifts vary from +13 nm (at *L* = 80 μm) to +48 nm (at *L* = 30 μm), showing up to 9% increase in the lattice layer spacing, which can be compared to the 17% increase seen in the SEMs (Figure 2b). We suspect that the lower layers of the opal are simultaneously compressed by the extrusion flow from under the posts, producing the blue-shifted reflection peaks which die away at higher flows (smaller *L*) as their order becomes worse. This contrasts with the smaller blue-shifts seen in the directly imprinted regions (III), from –5 nm (at *L* = 30 μm) to completely disappearing for *L* = 80 μm.

**Figure 5:**
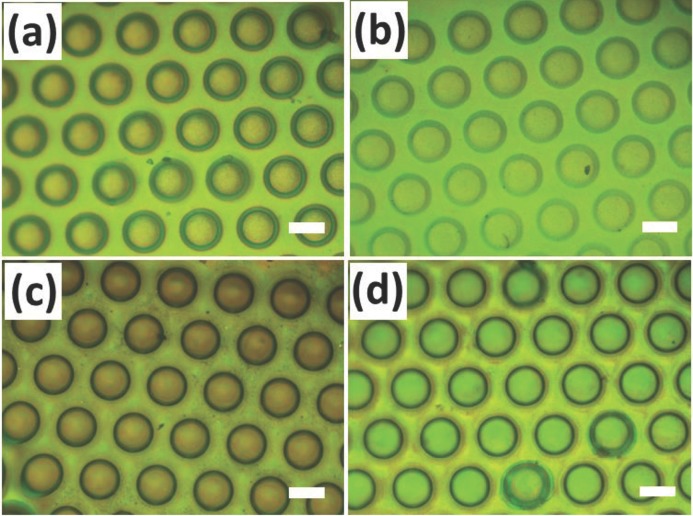
Optical images of adjustable structural color patterns under different imprinting conditions. (a) 110 °C, 10 bar, 150 s, (b) 90 °C, 30 bar, 150 s, (c) 110 °C 50 bar, 150 s, (d) 110 °C, 30 bar, 60 s. All of them have the same gap width of 80 μm as in Figure 4a. All scale bars are 100 μm.

According to Equation (1), the shear and extrusion flows of the hard particle cores and soft polymer shells are also dependent on the pressure, temperature and imprinting duration. Hence we can tune the color patterns by adjusting imprint pressure, imprint temperature which changes *η*(*T*), or through imprint duration. Lower imprinting pressures (10 bar) or lower imprinting temperatures (90 °C) result in a weaker flow of polymer and therefore less change in the colors (**Figure** **5**a, b). On the other hand, higher pressures cause red-shifts of the extruded regions but also disorder the directly imprinted region to destroy all structural color (Figure 5c). Shortening the imprinting duration results in shallower imprinting depths and reduced extrusion flow although the structural color of the imprinted region is still maintained (Figure 5d) unlike the situation in which it is destroyed (Figure 4a, III). The parameters introduced initially are thus near-optimal for producing strong color patterns on these POFs, but will change with viscosity and fill fraction of the polymer opals used.

### 2.4. Fine-tuning of Micropatterns by Stretching

**Figure 6:**
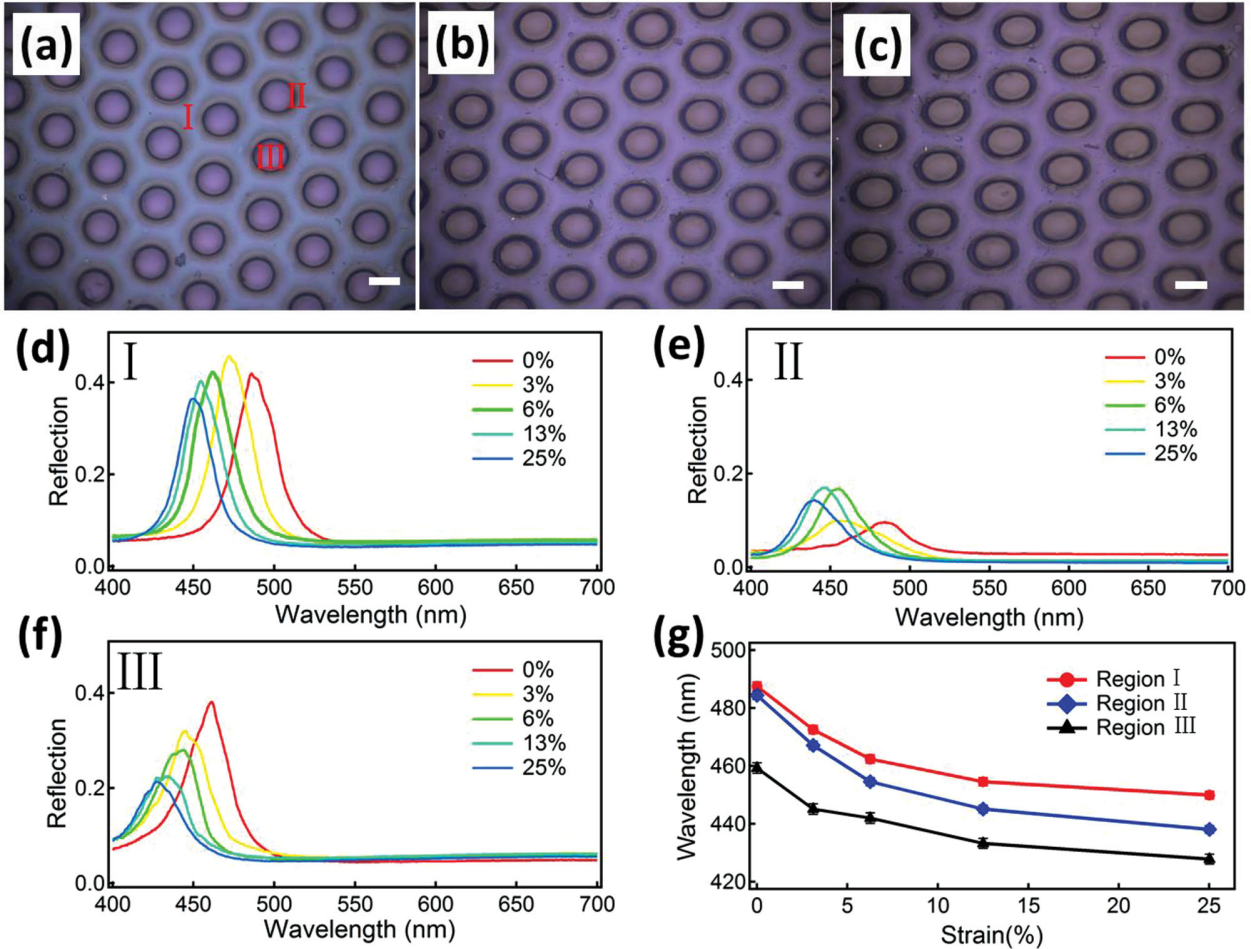
(a–c) Optical images of patterned POFs made of SiO_2_@PMMA@PEA at different stretch ratios up to 25% and (d–f) corresponding reflection spectra in three typical regions. (g) shows tuning of wavelength with strain. Scale bars in (a–c) are 100 μm.

**Figure 7:**
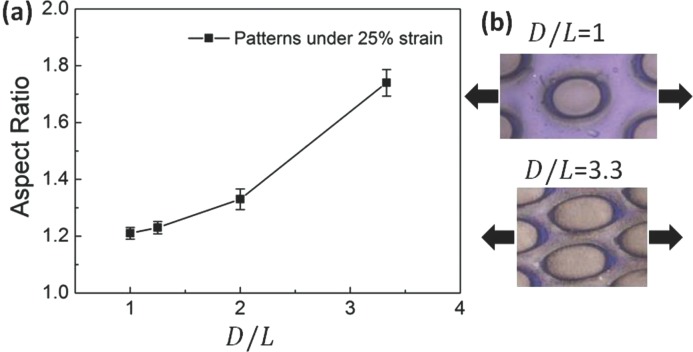
**(**a**)** Dependence of aspect ratio of the elliptical mesas on *D/L*, under strain of 25%. *D* is initial diameter of holes and *L* is edge to edge distance between the holes. (b) Local elasto-optics of films with different *D/L* = 1, 3.3 under equal 25% strain.

The structural properties of these POFs are very robust, flexible and elastic, and thus they can be strongly bent and stretched without deteriorating color quality.[28] This allows the active tuning of structural colors from these micropatterns by additional mechanical stretching, transforming the initial geometry of the patterns (**Figure** **6**). With increasing strain from 0% to 25%, circular holes gradually elongate uniaxially into ellipses and the color of the patterns monotonically blue-shifts. However due to the patterning, different regions blue-shift by different amounts at the same strain (Figure 6d-f from regions marked I, II, III in Figure 6a). These vary from - 31 nm under the posts, - 37 nm in between posts, to - 46 nm in the most sheared regions as indicated in Figure 6g. These blue-shifts are produced from the in-plane strain which induces a corresponding decrease of the lattice spacing in the normal direction (see detailed explanation in SI Figure S5). The imprinting geometry also modifies the shape deformations under strain, for instance changing with separation distance between posts (see SI Figure S6). As the separation distance decreases, the resulting aspect ratio of the elliptical holes produced under 25% strain increases (**Figure 7**). Thinner walls between the circular pits are easier to deform producing a greater anisotropy in the elastomeric response (Figure 7b). The POF system allows these inhomogeneous strains to be simply read out through a color image. Changing the pattern imprinted into these photonic crystals thus allows strain to be concentrated into particular regions, and thus to produce color changing patterns with intricate and optimized properties. These can be utilized in a variety of devices including strain sensors in applications from the built environment to wound healing.

## 3. Conclusions

In this paper we report a simple approach for large-scale patterning of photonic crystals using a microimprint technique. Polymer opal films with different initial ordering are microimprinted with stamps of posts under normal pressure and relatively low temperature. Because of the shear forces applied by the intrusion of the pre-patterned stamps, the soft PEA matrix in different regions presents different flow behaviors, resulting in different rearrangements of the hard spherical particles which produce the structural color. Lateral extrusion from under the posts leads to expansion of the spacing between neighboring layers in the region between the posts resulting in red-shifts of the structural color. Compression under the posts leads to a decreasing distance between neighboring layers, resulting in blue-shifts of the structural color. Around the post edges, strong shear forces produce distortions and defects in the lattice inducing a rapid loss of the reflection intensity. However we also showed that it was possible to use these local shearing forces to order random dispersions of spherical particles generating structural color patterns directly. Since the POFs are elastically deformable, the shapes of the imprinted pattern structures and their structural colors can be further tuned in laterally controllable ways by stretching. This simple and robust production of tunable photonic crystal micropatterns paves the way for further applications in optics, sensing, image displays and anti-counterfeit materials.

## 4. Experimental Section

*Fabrication of Polymer Opal Films*: The polymer opal films are fabricated according to previous reports,[27] but with improved ordering. The solution-grown colloidal aggregates of either PS@PMMA@PEA or SiO_2_@PMMA@PEA, are extruded into 1 cm-wide ribbons from a mini-extruder. This rubbery ribbon is cut into small segments and sandwiched between two PET films, followed by a rolling process to produce thinner wider films, which form the base unordered POFs. After a continuous shear-ordering process is then performed, the peak reflection intensity of the polymer opal films can be larger than 40%, resulting in the ordered POFs. One of the PET films is peeled off from the polymer opal surface to allow the microimprinting process.

*Microimprinting of Polymer Opal Films*: The stamps used for microimprinting were made by replicating PDMS molds with epoxy resin (Sigma-Aldrich Epoxy Embedding Medium Kit). The stamps are compressed against the polymer opal films at a pressure of 30 bar at 110 °C for 150 s using an Obducat Nanoimprinter. The imprinted polymer opal films are further cross-linked under UV to facilitate the de-molding process.

*Characterization*: Optical images of the micropatterned polymer opal films were recorded with an optical microscope (Olympus, BX51) and SEM images were taken with a Zeiss SEM at an accelerating voltage of 5 kV. Reflection spectra at different locations of the micropatterns were measured with an optical fiber spectrometer (QE6500, Ocean Optics) coupled to a confocal optical microscope, producing spectra from a 1 μm spatial spot. The selection of individual regions was monitored with the aid of the optical microscope.

## Supporting Information

As a service to our authors and readers, this journal provides supporting information supplied by the authors. Such materials are peer reviewed and may be re-organized for online delivery, but are not copy-edited or typeset. Technical support issues arising from supporting information (other than missing files) should be addressed to the authors.
