# Supplementary material for: Selectively Patterning Polymer Opal Films via Microimprint Lithography
Source: Adv Opt Mater. 2014 Sep 1;2(11):1098–104. doi: 10.1002/adom.201400327 (PMC4497474; doi:10.1002/adom.201400327)
Supplement: Supplementary file 1 — Supplementary [file adom0002-1098-sd1.pdf]

Copyright WILEY-VCH Verlag GmbH & Co. KGaA, 69469 Weinheim, Germany, 2014.

ADVANCED OPTICAL MATERIALS

Supporting Information

for *Adv. Mater.,* DOI: 10.1002/adom.201400327

Selectively Patterning Polymer Opal Films via Microimprint Lithography

*Tao Ding, Qibin Zhao, Stoyan K. Smoukov, and Jeremy J. Baumberg**

Copyright WILEY-VCH Verlag GmbH & Co. KGaA, 69469 Weinheim, Germany, 2013.

Supporting Information

Selectively patterning polymer opal films via microimprint lithography

*Tao Ding, Qibin Zhao, Stoyan K. Smoukov and Jeremy J. Baumberg**

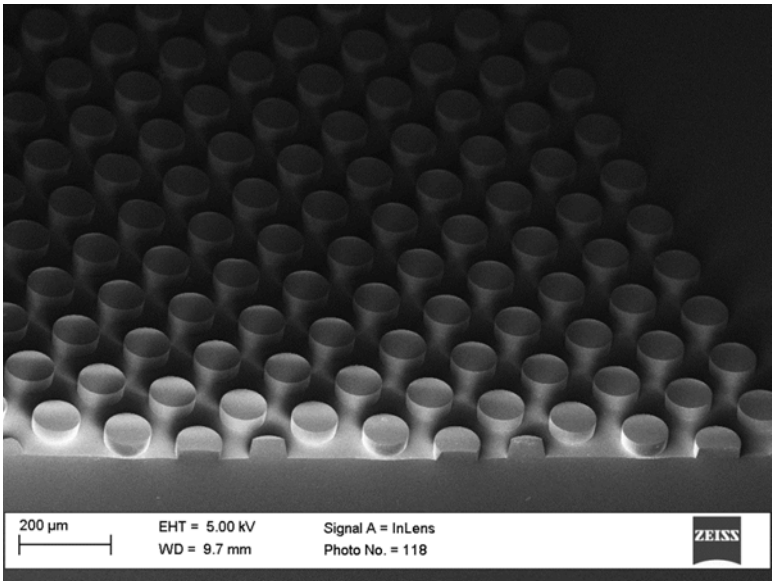

Figure S1. SEM image of resin stamp for microimprint lithography.

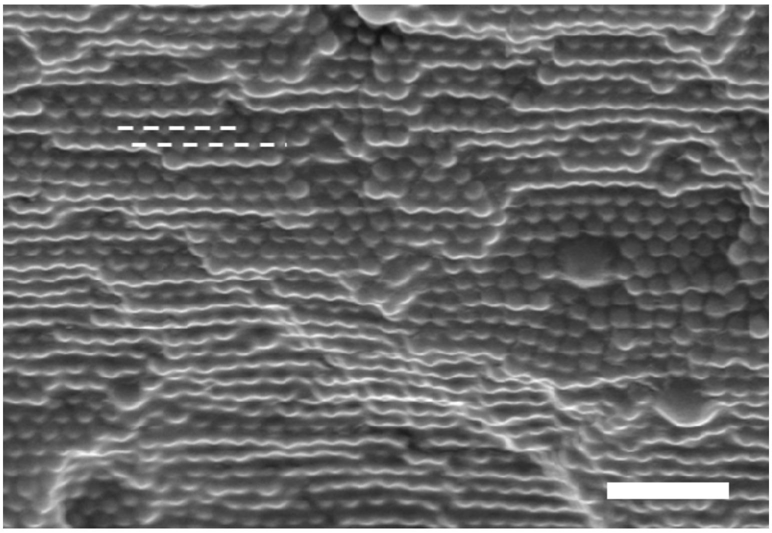

Figure S2. Cross section SEM image of SiO_2@PMMA@PEA POFs showing lattice distance around 128 nm. Scale bar is 1 µm

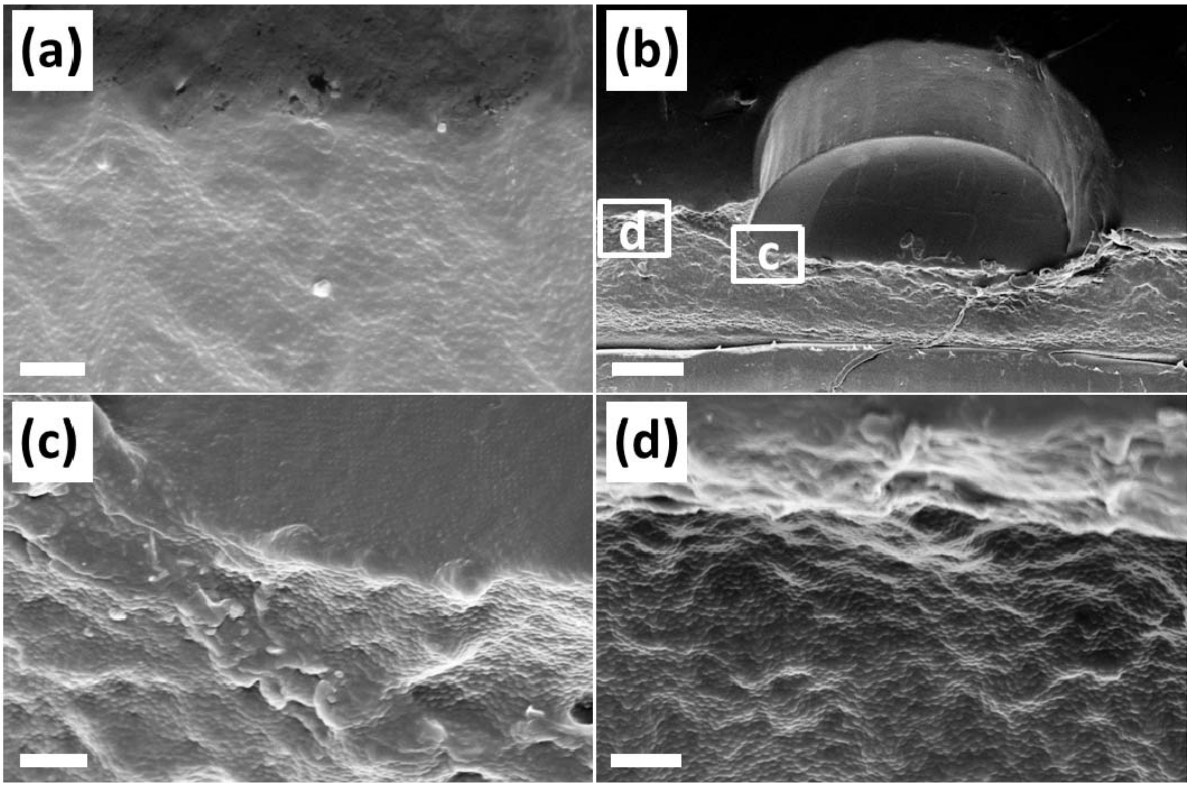

Figure S3. SEM images of unordered POFs (a) before and (b-d) after imprinting. (c) and (d) are the magnified view of imprinted and unimprinted regions whiteframed in (b). Scale bars in (a, c, d) are 2 μm and in (b) is 20 μm.

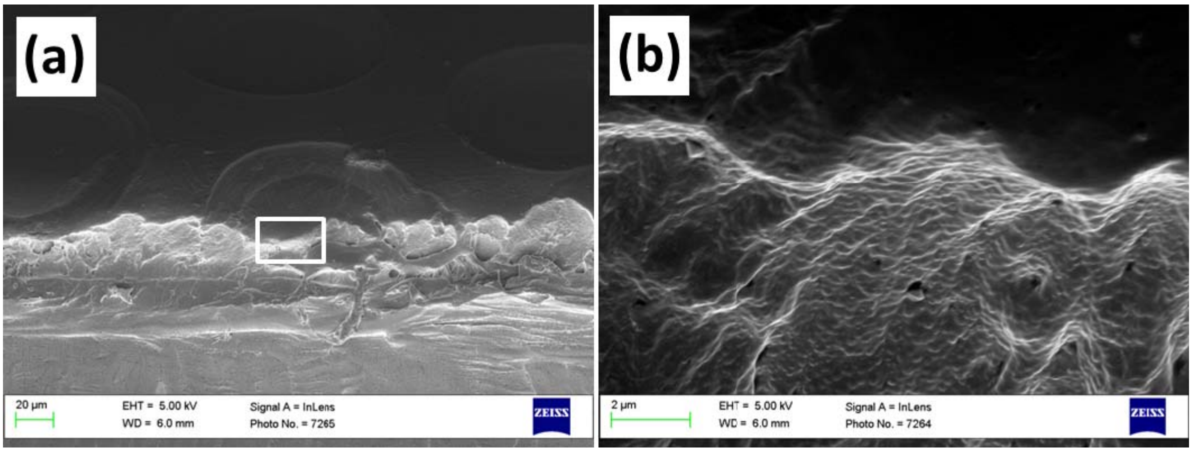

Figure S4. SEM images of POFs imprinted with stamp of post seperation L=80 μm. (b) is the magnified view of the imprinted regions showing deteriorated ordering. Scalr bar in (a) is 20 μm and in (b) is 2 μm.

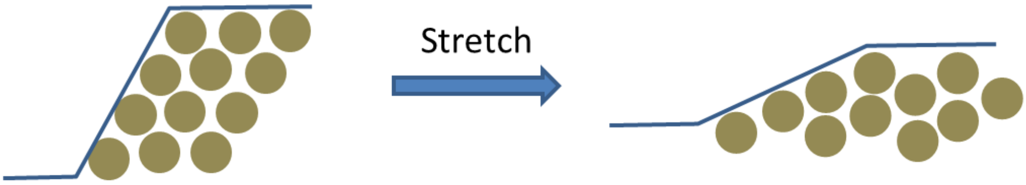

Figure S5. The scheme showing the change of incidence angle at the transition region while stretching.

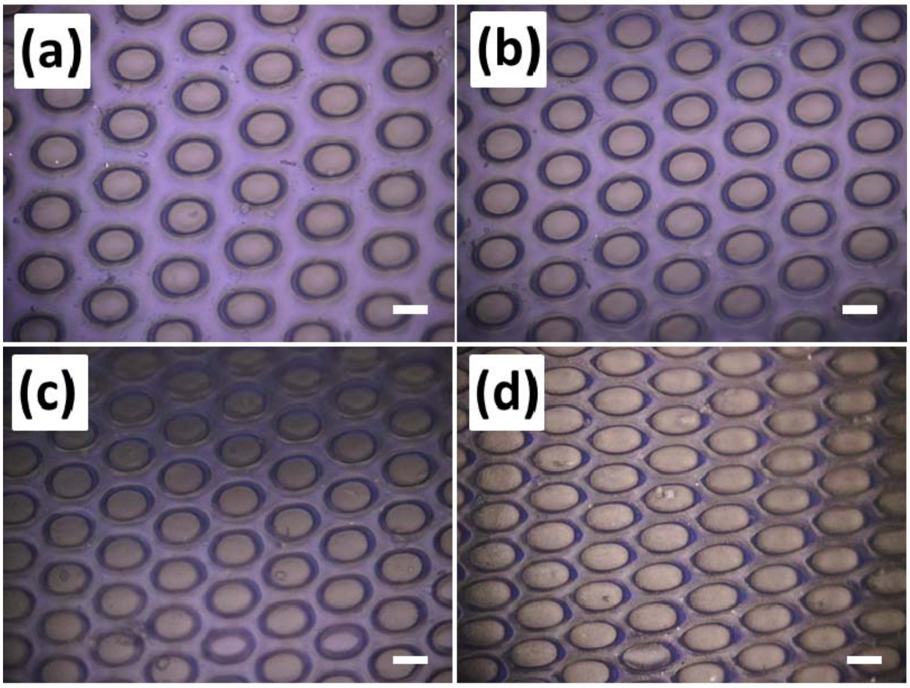

Figure S6. Optical images of uniaxially stretched micropatterns of POFs made of SiO_2@PMMA@PEA. The strain is 25% and separation between initially imprinted holes is (a) 100 μm, (b) 80μm, (c) 50 μm, and (d) 30 μm. All scale bars are 100 μm.

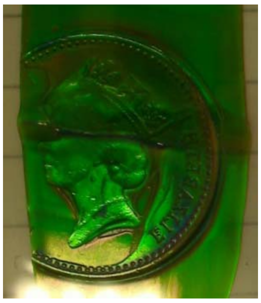

Figure S7. Large area image patterned POFs obtained by using a coin as the stamp.
